# Barriers and facilitators of public transport use among people with disabilities: a scoping review

**DOI:** 10.3389/fresc.2023.1336514

**Published:** 2024-01-08

**Authors:** C. R. Mwaka, K. L. Best, C. Cunningham, M. Gagnon, F. Routhier

**Affiliations:** ^1^ Center for Interdisciplinary Research in Rehabilitation and Social Integration, Centre Intégré Universitaire de Santé et de Services Sociaux de la Capitale-Nationale, Québec, QC, Canada; ^2^ Faculty of Medicine, School of Rehabilitation Sciences, Université Laval, Québec, QC, Canada; ^3^ Department of Medicine, Faculty of Medicine, Université Laval, Québec, QC, Canada; ^4^ Library, Université Laval, Québec, QC, Canada

**Keywords:** public transport, accessibility, disability, self-efficacy, satisfaction, scoping review

## Abstract

Barriers to public transport use may be experienced differently by people with various types of disabilities (e.g., physical, intellectual, cognitive, sensory). Thus, it is important to identify the variable needs within each element of the travel chain. For example, the unavailability or low volume of auditory announcements in a stop or station or on the public transport vehicle may be a barrier to people with visual disability who rely on hearing the information. Consequently, this could provoke negative emotions and unpleasant experiences, which may not be the case for people with physical disabilities. The primary objective was to describe the barriers and facilitators to using public transport experienced by people with disabilities (PWD). The secondary aim was to explore experiences in terms of self-efficacy and satisfaction, when using public transport among people with disabilities. A scoping review was conducted. The search was performed in MEDLINE, TRANSPORT DATABASE, PsycINFO, EMBASE, and WEB OF SCIENCE from 1995 to 2023. Of 6,820 citations identified, 34 articles were included in the review for extraction. The main physical and social barriers included lack of ramp, long walking distance, long waiting time, unavailability of information at bus stop or station, and drivers' negative attitudes towards PWD. Personal factors that prevented the use of public transport included lack of confidence, and decreased satisfaction with public transport use. Strategies such as providing ramps on public transport vehicles, availability of kneeling buses and courtesy of bus drivers, and travel training were considered as enablers to the use of public transport that can lead the improved self-efficacy and satisfaction. In conclusion, this review identified the physical and social barriers and facilitators in travel chain, and highlighted issues related to lack of confidence or self-efficacy and decreased satisfaction when PWD and older adults are using public transport. Identifying and understanding the barriers and facilitators to the use of public transport by PWD is a milestone that may help policy makers and transport operators around the world to develop and implement interventions enabling access, use and inclusion of this mode of transport, as the experiences of PWD when using this mode of transport have an impact on their well-being.

## Introduction

The United Nations Sustainable Develop Goals and the 2030 Agenda for Sustainable Development emphasizes the importance of providing accessible and sustainable transportation systems for all citizens, including the development of public transport, while paying particular attention to the needs of people in vulnerable situations such as people with disabilities (PWD) (1). In this instance, disability refers to the interaction between individuals with a health condition (could be physical or mental) with personal and environmental factors including negative attitudes, inaccessible transportation and public buildings, and limited social support (2). Complementary, universal design is intended to ensure the design and composition of an environment is achieved in such a way that it can be accessed, understood and used to the greatest extent possible by all people, regardless of age, size, or ability (3). Thus, when designing and planning public transport, it is critical to consider accessibility and how to meet the needs of all potential users. Without accessible public transport options, PWD may not be able to easily leave their homes, thus may incur extra expenses to accessing basic community services (4) and are at higher risk of isolation (5). Accessible public transport can facilitate autonomy for PWD, by proving access to community-based services and meaningful social roles, at convenient times. However, to be accessible, PWD must be informed about public transport services, have adequate knowledge, be able to use public transport services, and to be able to afford public transport services (6). Despite the adoption of the United Nation (UN) Convention on the Rights of Persons with Disabilities (UN-CRDP) (7) and efforts to facilitate access to and use of public transport by PWD, many physical and social barriers remain.

Public transport is defined as a system of vehicles, such as buses and trains, that operate at regular times on fixed routes and are used by the public (8). Public transport can play an important role in the travel chain, which suggests that any given travel starts at the origin of the users (e.g., their home) and ends at the final destination (9). In this way, important links of the travel chain include leaving the home to wait for the transport at the stop or station, availability of timetable information, boarding, moving within the transport, disembarking, the use of sidewalks, and the attitudes of drivers and other passengers toward PWD (9).

Barriers and facilitators to the use of public transport may be experienced at any link in the travel chain by PWD. Given the complex and multiple steps required to use public transit, the entire travel chain must be considered to adequately accommodate PWD. It is therefore important to consider leaving how PWD leave the home and get to the stop or station, waiting times, availability of timetable information, boarding, moving within the transport, disembarking, the use of sidewalks, the attitudes of drivers and other passengers towards PWD (10). If there are missing links, experiences with public transport use will likely be less than satisfactory. Various barriers and facilitators can affect self-efficacy and satisfaction with public transport use among PWD, and thus willingness to use public transport (11).

Self-efficacy, defined as belief in one's ability to perform a specific task (12), is considered the most important predictor of travel behavior change (13). Self-efficacy is influenced by factors including past experiences or accomplishments and emotional reactions (12–15). Past accomplishments that are interpreted as the result of a skill developed in the past (16) have been found to be the most influential in influencing self-efficacy (15). Emotional reaction can improve or reduce self-efficacy. Thus, positive experiences with public transport use may generate positive emotions that can enhance feelings of personal efficacy toward public transport use. Conversely, negative experiences can induce negative emotional reactions such as anxiety and doubt, which in turn may impact self-efficacy for using public transport. Therefore, low self-efficacy may be a barrier for public transport use (13).

In two systematic reviews, Risser et al. (17) and Unsworth et al. (18) described public transport accessibility for people with cognitive and mobility impairments respectively. Barriers identified in the selected studies included lack of assistive devices and trained personnel to assist with orientation, problems related to orientation and navigation, uneven pavement, lack of curb-cuts, stairs, narrow doorways, high placement of controls for pedestrian lights and elevators, poor design of street signs, information placed out of reach/sight, inappropriate spaces for wheeled mobility devices, lengthy wait times, and inadequate shelters. However, these studies did not include other types of disabilities, such as visual, hearing, autism, mental/intellectual disabilities, findings cannot be generalized to all PWD.

Given that barriers to public transport use may be perceived and experienced differently by people with various types of disabilities (e.g., physical, intellectual, cognitive, sensory), it is important to identify the variable needs within each element of the travel chain. For example, the unavailability or low volume of auditory announcements in a stop or station or on the public transport vehicle may be a barrier to people with visual disability who rely on hearing the information. Consequently, this could provoke negative emotions and unpleasant experiences, which may not be the case for people with physical disabilities. Similarly, a person with a physical disability who uses a manual wheelchair may have difficulty boarding and the bus in the absence of a ramp, yet a person with a hearing disability may not experience the lack of ramps as a barrier.

Satisfying experiences tend to increase intrinsic motivation, which increases the likelihood to continue a given behavior. Consequently, experiencing satisfaction [i.e., intrinsic positive consequence emerging from a behavior that fulfills the expectations of an individual (19)] during a given activity increases the likelihood for sustained behavior change (13). The most relevant features of the transportation system, such as trip duration, accessibility, fare, network connectivity, information, comfort, safety, and kindness of employees, may influence user satisfaction. Satisfaction with travel can have a significantly positive effect on the frequency of public transport use (20). Indeed, the more satisfied public transit users are with their travel experience, the more they tend to use public transport for their work commute (20).

To respond the gap in the existing literature, this study first aims to examine barriers and facilitators to the use of the public transport (buses, trains, tramway, ferries) by people with different types of disabilities during the entire travel chain. Secondly, this study aims to explore perceived self-efficacy and satisfaction related to public transport experiences among PWD.

## Method

We conducted a scoping review to examine the extent of research activity related to the barriers and facilitators experienced by PWD, and their perceived feelings of self-efficacy and satisfaction when using public transportation. The methodology and results were reported according to the Preferred Reporting Items for Systematic Reviews and Meta-Analyses Extension for Scoping Reviews (PRISMA) checklist (21). Arksey and O'Malley's methodological framework guided the review through five stages: (1) identifying the research question; (2) identifying relevant studies; (3) study selection; (4) charting the data; and (5) collating, summarizing, and reporting the results (22). The literature search was conducted in 5 relevant databases including MEDLINE, TRANSPORT DATABASE, PsycINFO (from Ovid platform), Embase, and Web of Science from January 1995 to July 2022, and update was made in May 2023. Studies meeting the eligibility criteria presented in Table 1 were included in this review for date extraction. The results are will be described according to the Human Development Model-Disability Creation Process (HDM-DCP) conceptual framework (23) with respect to the research questions, and the aims of this scoping review. The HDM-DCP conceptual framework addresses disability situations that can arise when personal and environmental factors restrict life habits, thus reducing social participation. It includes personal (e.g., disability) and environmental (e.g., physical, or social barriers/facilitators) factors, and life habits (e.g., the public transport use to go to work). Full details on the development of the methodology, and registration are provided in a published protocol of this scoping review (24).

**Table 1:** Inclusion and exclusion criteria.

Article type	
Inclusion criteria • Original peer-reviewed manuscript • Guideline report • Concerned with public transport (bus, train, tramway, ferry) accessibility or barriers/facilitators or confidence and satisfaction with public transport for people with disabilities including older people • Published from January 1995 to July 2022 • Published in English or French	Exclusion criteria • Article for which the full text is not available • Article focuses on the use of technology while using public transport system • Article generally discuss universal design or transportation • Validation study of measurement tools assessing public transport accessibility or travel confidence or travel satisfaction for people with disabilities • Study not involving fixed route public transport (e.g., adapted school bus for students with disabilities, paratransit) • Protocol on public transport accessibility for PWD

## Results

**Figure 1:**
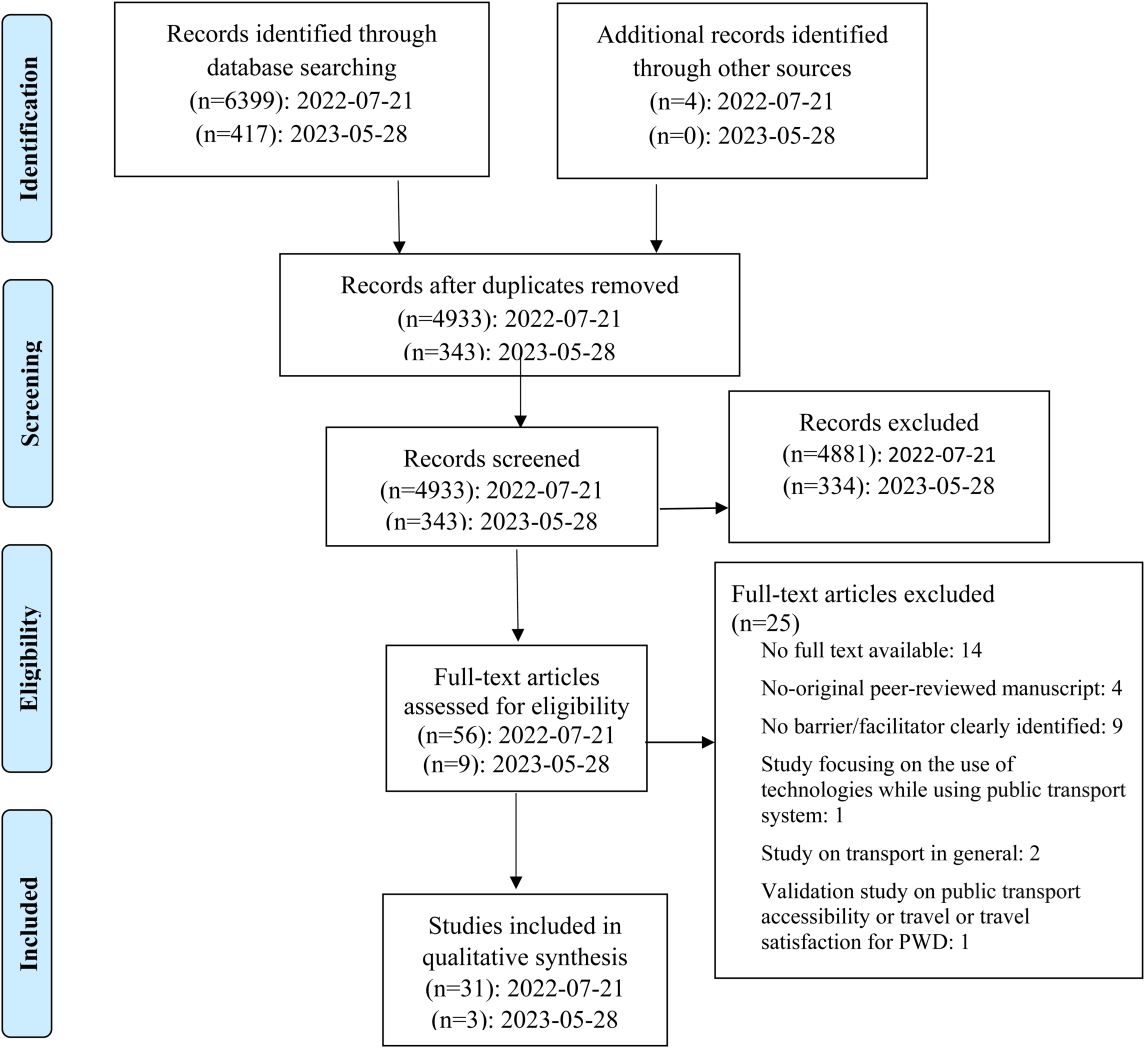
PRIMSA flowchart.

As shown the PRIMSA flowchart (Figure 1), a total of 6,816 citations were retrieved in the databases targeted by our research performed in July 2022 (*n* = 6,399) with the search updated in May 2023 (*n* = 417). Four additional references were also hand-selected from the references lists of two systematic reviews (17, 18) for initial screening. After removing duplicates, 5,276 titles and abstracts were screened, and 65 full-text articles were reviewed. Finally, 34 studies met inclusion and exclusion criteria for data extraction.

The age of participants in the 34 selected studies ranged from 12 to 77 years old, and most of them had physical, visual, auditory, intellectual, and mental disabilities, and speech conditions. Of these studies, 19 focused exclusively on PWD, 10 involved older adults, 3 focused on PWD and healthy people, and 2 involved PWD and older adults (Supplementary Table S2). With regards to infrastructure or fixed-route transit mode used, 12 studies focused only on buses, 7 on buses and trains, 3 on buses and rails, 1 on bus and bus stop, 1 on metro stations, and 9 studies combined several modes of public transport including boats, buses, trains, trams, light rails, subway, planes, streetcars, online taxi, private car, and motorcycle (Supplementary Table S2).

Regarding the study design of included studies, Seventeen studies (25–41) used a qualitative cross-sectional design, six (11, 42–46) used quantitative cross-sectional design, three (47–49) were cross-sectional mixed methods, two (50, 51) were longitudinal pre-post quantitative studies, two (52, 53) used an explanatory descriptive approach, one (54) used a longitudinal mixed method, another one (55) used multimethod approach, and two (17, 18) were systematic reviews (Supplementary Table S2). In terms of barriers or facilitators that influenced public transport use, eighteen studies (11, 17, 18, 25–27, 29, 33, 36–38, 40, 43, 46, 48–50, 54) reported both physical and social barriers and facilitators (e.g., lack of ramp or concerns with ramp angle and deployment, lack of training of drivers and other systems users on the needs of PWD, resulting in lack of respect, free pass, lower floor buses, mobility training for people with visual impairment and PWD/age-awareness training for bus drivers), two (31, 55) reported physical barriers (e.g., winter, ice, snow) and personal factors (e.g., fear of injury, lack of knowledge, or self-efficacy), two (11, 35) described only physical barriers, four (30, 39, 42, 45) reported physical and social barriers and facilitators along with influencing personal factors (e.g., fear of being harassed inside the crowded buses, lack of knowledge of the public transport system), six (29, 33, 45, 46, 52, 53) described user satisfaction only, and one (51) described self-efficacy (Supplementary Table S2). Specific details of all barriers, facilitators and influencing factors are presented in Supplementary Table S2.

Several barriers and facilitators, and perceptions in terms of self-efficacy and satisfaction when using public transport among PWD were described in Supplementary Table S2. The overview of results of this review was organized according to various links of the travel chain, describing the presence of physical and social barriers and facilitators, as well as personal factors including self-efficacy and satisfaction, and the HDM-DCP conceptual framework.

### Travelling to or from the public transport stop or station

Fifteen studies (16–18, 26, 28, 32, 33, 36, 38–40, 44, 47, 50, 54) reported physical barriers faced by PWD in getting to and from public transit stops or station. These barriers included long walking distance, irregular walking surface, narrow pathways, branches hanging in pathways, small holes, poor design of curb cuts, difference in levels, steep side gradient on pathways, low contrast in surface changes, combined pedestrian and bike lanes, grey posts on pathways, crossing with traffic light but no auditory signal, too short time for walk lights, slippery white lines, traffic from two directions, turnstiles lack sound modules to provide information about remaining balance on travel passes, crossing busy streets, lack of sidewalks, weather conditions (e.g., snow, ice and rain, and wind), darkness and unlighted areas, road work, lack of pavement, and lack of dropped curbs.

Considering the physical barriers that may be encountered during this link of the travel chain, five studies (31, 38, 39, 50, 54) have proposed factors which may facilitate access to the physical environment, such as improved pedestrian infrastructure, touch elements in signals, turnstiles with sound modules to assist people with visual impairments, adapt ticket booths and turnstiles for users of different heights, safer intersections with traffic-calming measures, improved infrastructures with tactile guidance (e.g., floor tiles with gradient textures), snow removal, and public transport route planning with common destinations located short walking distances from bus stops, and the use of visual or audible cues. No studies identified social barriers when going to or from the public transport stop or station.

### Waiting at the stop or station

Fourteen studies (18, 28, 30, 36–40, 42, 43, 46, 47, 49, 54) have documented physical barriers experiences while waiting at the stop or station. These issues included unavailable information in terminal or bus stop, drivers not stopping to let people board the bus, platform design and lack of signage, signage quite high and far, signage to bright with glare, high levels of noise, lack of visual announcements on the train, narrow bus stops, grass on bus stop, no weather protection or shelters, no seats or inadequate seats (too high, too low, without back rests), back of the seat slopes backwards, many buses stop at the same bus stop, lack of timetable, small text on timetables, poor visibility on monitors, wrong information, difficult to interpret information, information table too far away, no information about routes in service, no information provided in braille, presence of stairs in railway stations, insufficient lighting in stations, unclean bus-stops and train stations and frequently occupied by people begging, broken elevators or escalators, and long wait times. Indeed, of thirty-one studies reviewed, three reported that long waiting times at bus stops were a barrier to the use of public transport by PWD (18, 30, 47).

The following facilitators to improve access to bus stops or stations have also been identified: touch elements in signals, turnstiles with sound modules for people with visual disabilities, ensuring seating places at bus stops for older adults, making electronic display information of bus arrival/bus delay available, installation of guiding blocks in the railway stations, stairs, and move level surfaces (38–40, 50). One study identified identification of bus numbers as a specific personal factor for people with sight loss (27).

### Boarding and getting of the public transport

Nine studies (18, 26, 29, 38, 42, 43, 46, 48, 54) focused on physical barriers related to ramps, including lack of ramp, inoperable ramps, steep slope for ramp use, and ramp deployment angle (≥9.5°). These barriers were experienced especially by wheelchair users. Concerning the deployment of the ramp, American Disability Act (ADA) proposes a maximum allowable ramp slope of 9.5°. Six studies (18, 26, 36, 37, 39, 43) identified the presence of steps at the vehicle entrance as barrier occurring when PWD are boarding or getting off the public transport. Social barriers included false claims of inoperable lifts or ramps made by drivers to avoid letting a PWD board, stress generated by social expectation to be quick as healthy persons, some bus drivers that do not deploy ramps, others do not kneel the buses for unknown reasons were reported in five studies (26, 36, 38, 43, 54). Furthermore, four studies (18, 30, 42, 50) explored factors facilitating access to public transport, including providing ramps on public transport vehicles, availability of kneeling buses and courtesy of bus drivers to lower the bus floor to facilitate boarding (34).

### Within the public transport vehicle

Ten studies (18, 25, 26, 29, 30, 37, 38, 40, 47, 54) focused on the physical barriers within the public transport vehicles. These barriers included lack of space or less space for accessible seating, seats sloped backwards, seats without back rests, bus doors swung out, makes stops at unmarked bus stops, all seats occupied, no handrails at seats, narrow spaces between seats, narrow spaces to the seat in front, standing on a bus while it is in motion, wrong information on bus stop information, lack of space for circulation, lack of reliable audible announcements on trains and buses, alert buttons too high, unavailability of seatbelts to secure wheelchair users in place, seats too low, inadequate amount and indication of priority seats. Four other studies attempted to understand the components of the enabling physical environment encountered by PWD on public transport (17, 30, 34, 37, 42). Physical enablers included more space at PWD seats, and grab rails priority seating for older adults, making visual and availability of auditory announcements in the buses, lower pull-cords to call stops, reliable information during the trip.

Ten other studies (29, 33, 36–38, 40, 41, 43, 46, 54) identified social barriers such as less assistance by staff, barriers closely relate to problems with fare cost, lack of respect and buses drivers' behavior causes sudden brakes and acceleration causing discomfort to users, drivers not calling out stops, concerns related to timing and safety, reliability concerns, difficulty to access and exit because of crowdedness, drivers not calling out stops, greater service animal issues for people with loss of sight, unwanted physical assistance and verbal and sexual assault, lack of education of other passengers about health concerns that PWD can have, judgmental and reductive comments made by young passengers towards PWD, safety problems, lack of training of drivers and other systems users on the needs of PWD, drivers stop vehicles far from the platform or bus stop, conflict between wheelchair user and parents with buggies onboard the bus, lack of knowledge for staff on the use of ramp access and other needs of PWD, lack of courtesy from drivers, lack of confidence in the staff (11, 18, 25, 29, 30, 37–42, 44, 46, 54). Eight studies (18, 30, 36–39, 46), reported negative attitudes of buses drivers without specifying the type. Other rare research (29, 36, 49) investigated social enablers such as improved behavior at doors for passenger entrance and exit, reducing bus and train occupancy levels, and adaptation and enforcement of use of preferential spaces, friendly and courteous bus drivers; social interaction by meeting new people on the bus, discounted senior or PWD fares. The resolution of these social problems occurring within public transport requires the involvement of transport service providers and government authorities. Transport service providers should train and educate drivers about their behaviour towards PWD. Government authorities should consider the fact that PWD and older people generally have low incomes and therefore provide subsidies or exemptions from certain charges (e.g., taxes) to transport service providers so that they, in turn, can commit to providing an affordable preferential fare. This can help make public transport not only accessible, but also inclusive and usable.

### Other issues related to public transport use

Twelve studies (18, 27, 31, 35, 38–40, 42–44, 51, 55) reported personal factors such as other issues related to the use of public transport, including inability to navigate public system, lack of confidence in the use of public transport, lack of knowledge of public transport network, and fear of injury related to public transport.

## Discussion

This review examined barriers and facilitators encountered by PWD and highlighted their perceived experiences in terms of self-efficacy and satisfaction from 1992 to 2023. Being able to travel by public transport modes such as bus, train, air or ship is an expression of autonomy and facilitates social interactions (42). However, PWD are likely to often encounter difficulties using public transport in their daily lives due to widespread physical and social barriers. Bezyak et al. (43) argued that, despite removal of many physical barriers within fixed-route systems, significant barriers to overall access of public transit systems are still present. This is all the more obvious as the results of this review show that 85.3% (*n* = 29) of the identified studies which pointed out real and perceived barriers of transit-related use and the resulting feeling of dissatisfaction were conducted after 2006, corresponding to the year of adoption of the UN-CRPD [5]. This implies that, despite efforts in terms of legislation, development and implementation of access measures, many physical and social barriers to accessing and using public transport remain and prevent PWD from carrying out many of their life habits activities.

Our study has highlighted the physical barriers that PWD and older adults experienced when travelling to and from stops or stations. These barriers included long walking distance, irregular walking surface, narrow pathways, etc. Travelling to or from stops or stations can be influenced by the characteristics of the built environment, such as the condition of roads and sidewalks, safety, lighting, and the distance between home or another benchmark (e.g., school or market) and the stop. This Particularly regarding walking distance between a benchmark and a stop, UN habitat considers that access to public transport is considered appropriate when a stop is accessible within a walkable distance along the street network of 500 m from a reference point such as a home, school, workplace, market, etc. to a low-capacity public transport system (e.g., bus, Bus Rapid Transit) and 1 km to a high-capacity system (e.g., rail, metro, ferry) (56). Walking distance has been shown to be an important predictor of the frequency of public transport use (44, 57). It is the most important factor to consider when travelling to or from stop or station for at least two reasons: walking is the primary access mode for trips from home to public transit and walking distance has a significant impact on public transport use (57). And this seems all the more plausible given that of the fifteen studies that have reported on the physical barriers that occur when walking to or from a bus stop/station, eight (26, 28, 32, 33, 36, 44, 47, 50) have identified walking distance as a barrier to using public transport. Travelling to or from stop/station must be understood as an integral part of the travel chain, during which barriers may emerge and limit access to and use of public transit by PWD. Government authorities responsible for managing the city should implement measures to make the pedestrian environment accessible to PWD while reducing the home-stop/station walking distance in line with the UN-Habitat recommendations (56). Consistent with findings from Unsworth et al. (18), no studies identified social barriers when going to or from the public transport stop or station. Further research could be carried out to identify the social barriers likely to occur in this link of the travel chain and which may limit the use of public transit by PWD.

Another link in the travel chain where barriers such as the unavailability of travel information, drivers not stopping to let people board the bus, platform design and lack of signage, signage quite high and far, long waiting times, etc. have been identified is waiting at the stop or station. Of the fourteen studies that had identified barriers in this link, three (18, 30, 47) pointed out the long waiting time. Waiting time at a stop/station has been shown to be the temporal component of the travel to which passengers are most sensitive (58). Even a small increase in this time can significantly affect confidence and push them towards other modes of transport (59). UN-Habitat recommends that public transport should have no more of 30 min average waiting time during peak hours (from 5 am to 9 pm) to assess the frequency of the service (56). The responsibility for reducing barriers at bus stops or stations is shared between government authorities and public transport service providers. For example, government authorities are responsible for making bus stops or stations accessible, while the responsibility for reducing waiting times and improving access to information at bus stops lies with public transport service providers. Given we identified no study that has documented the social barriers at public transport stops or stations, research exploring social barriers and facilitators, including personal factors, is needed to guide government authorities and public transport service providers in how tin how to best respond to the public transport needs of PWD.

Nine (18, 26, 29, 38, 42, 43, 46, 48, 54) of the thirty-four studies included studies highlighted the physical barriers associated with ramp issues, and specifically concerned, for example lack ramp, inoperable ramps, steep slope for ramp use, and ramp deployment angle exceeding 9.5°, with as often associated social barriers such as claims inoperable lifts or ramps made by drivers to avoid letting a PWD board, bus drivers that do not deploy ramps or do not kneel the bus for unknown reasons (22–24, 34, 41). Concerning the deployment of the ramp, American Disability Act (ADA) proposes a maximum allowable ramp slope of 9.5°. Transport operators must ensure that ramp design and deployment features comply with ADA recommendations. Lenker et al. assert that the accessibility of access ramps is affected by their slope, which is often described by a ratio, a:b, indicating a rise of a inches for every b inches in run (60). On this basis, the ADA is recommending that ramps shall have the least slope practicable and shall not exceed 1:4 when deployed to ground level. If the height of the vehicle floor from which the ramp is deployed is 3 in or less above a 6-in curb, a maximum slope of 1:4 is permitted; if the height of the vehicle floor from which the ramp is deployed is 6 in or less, but greater than 3 in, above a 6-in curb, a maximum slope of 1:6 is permitted; if the height of the vehicle floor from which the ramp is deployed is 9 in or less, but greater than 6 in, above a 6-in curb, a maximum slope of 1:8 is permitted; if the height of the vehicle floor from which the ramp is deployed is greater than 9 in above a 6-in curb, a slope of 1:12 shall be achieved (61). This implies that it's not enough just to equip public transport vehicles with suitable ramps; drivers also need to know how to use them, so that they are not perceived by PWD as another source of difficulties preventing them from using this mode of transport. Furthermore, six studies (18, 26, 36, 37, 39, 43) underlined the presence of steps at the entrée of the public transport. PWD are considering steps as walls preventing them from using public transport. Transport operators are called upon to remove these steps to make public transport accessible and usable for this category of the population, which represents around 15% of the world's population.

This review also identified the barriers encountered inside the vehicle. These barriers include physical barriers such as lack of space or less space for accessible seating, seats sloped backwards, seats without back rests, bus doors swung out, makes stops at unmarked bus stops, all seats occupied, narrow spaces to the seat in front, lack of space for circulation, lack of reliable audible announcements on trains and buses, etc. Other barriers concerned social aspects included less assistance by staff, barriers closely relate to problems with fare cost, lack of respect and buses drivers' behavior causes sudden brakes and acceleration causing discomfort to users, drivers not calling out stops, verbal and sexual assault, lack of education of other passengers about health concerns of PWD. These barriers, like the others mentioned above, can have a negative impact on the quality of life and well-being of PWD, by contributing to limit their social inclusion and participation. It is the transport operator's responsibility to address these barriers within the public transport vehicle. The transport operators should have to ensure that all seats reserved for PWD are well adapted, with adequate space to facilitate handling and movement by technical aids. The transport operator is also responsible for making passengers and drivers aware of their attitudes towards PWD and the older adults. Six studies.

Beyond theses physical and social barriers, this study also underlined some personal factors which can limit access and use of public transport among PWD. These included lack of confidence or self-efficacy in the use of public transit (31, 36, 40, 51), decreased satisfaction (29, 45, 46), lack of knowledge of the public transit system (24, 32, 49), and fear of transit-related injuries (31, 35, 39), with special mention for the first two personal factors. Considered as the belief in one's ability to perform a specific task, self-efficacy has been shown to be the most important determinant of behavior change (13). Its reduction regarding public transport can be improved by travel training. Studies on travel training in the use of public transport for PWD and older adults show that such travel training helps them to improve their self-efficacy and knowledge in public transport system, and therefore to overcome fear of the use of public transit (51) and have a satisfactory travel experience. Rehabilitation professionals have a critical role to play in the process of developing training programs and learning how to use public transport for PWD, especially those with lack knowledge and low self-efficacy in their ability to use public transport.

Satisfaction with public transport is often associated with the quality of service provided by the transport operator. That said, if public transport is not accessible due to physical and social barriers, this can lead to a bitter and regrettable experience for PWD, and consequently affect their willingness to use this mode of transport. Conversely, an accessible public transport system can lead to a positive and satisfying experience that can improve the frequency of the use of this mode of transportation to fulfill life habits. The scientific literature argues that the more satisfied people are with their travel experience, the more they tend to use public transport for their work commute (20). Therefore, transport operators are called upon to improve the quality of the services they provide to PWD to promote their inclusion and social participation.

### Implications for rehabilitation

• Barriers and facilitators to public transport may be experienced differently by people with disabilities depending on their individual situation of disability (e.g., physical, intellectual, cognitive, visual, or hearing disability).
• Best-practices in public transport may be targeted towards transport providers and policy makers to make public transport accessible, usable, and inclusive for people with all types of disabilities.
• Modifications to the environment (e.g., ramps), and interventions (e.g., staff awareness and education, training in the use of public transport) may facilitate accessibility and use of public transport by people with disabilities.
• Improved public transport use may facilitate social inclusion, participation, and well-being for people with disabilities, the ultimate goal of rehabilitation.

### Strength and limits

The strength of this study lies in its methodology. Indeed, we developed this review based on Arskey and O’Malley's methodological framework. This methodological framework is widely used in the development of such a literature review. Moreover, we used a technical support of a librarian with expertise in the development of documentary research strategies applied to rehabilitation to ensure that we retrieved the maximum number of citations related to the topic of this scoping review. Furthermore, the screening of citations and the articles, and the extraction of data were carried out rigorously and independently by two reviewers to control biases related to any possible loss of information, yet relevant. However, this scoping review also has its limitations. First, even if the keywords used in the search strategy were broad, they might not identify all specialized studies in public transport accessibility for PWD despite consulting of librarian in the choice of keywords and the refinement of the search strategies. Moreover, the fact of having considered only English and French as the languages of publication excluded papers.

## Conclusion

This study shows that people with various forms of disability continue to encounter difficulties in accessing and using public transit throughout the entire travel chain, due to many physical and social barriers. despite the adoption and implementation of the CRDP. This review identified the physical and social barriers and facilitators that can occur in different links of the travel chain and highlighted issues related to lack of confidence and decreased d satisfaction when PWD and older adults are using public transport. The identification of barriers and facilitators to the use of public transport by PWD is an important step that may help policy makers and transport operators around the world to develop and implement interventions to facilitate access, use and inclusion of this mode of transport, as the experiences of PWD when using this mode of transport have an impact on their well-being. The results of this scoping review could lead to a better understanding of the potential barriers and facilitators to the use of public transport by people with various disabilities and how negative or positive experiences throughout the travel may influence their self-efficacy and satisfaction.
