# Supplementary material for: Barriers and facilitators of public transport use among people with disabilities: a scoping review
Source: Front Rehabil Sci. 2024 Jan 8;4:1336514. doi: 10.3389/fresc.2023.1336514 (PMC10812606; doi:10.3389/fresc.2023.1336514)
Supplement: Supplementary file 1 [file Table2.docx]

Supplementary materials

| **Table 2.** Characteristics of included studies | | | | | | |
| --- | --- | --- | --- | --- | --- | --- |
| **Author(s), year** | **City, Country** | **Sample (size; age; sex [%men]; population type; disability type)** | **Design** | **Infrastructure/mode of public transport used** | **Objective** | **Main outcomes** |
| Almada and Renner, 2015 (47) | Novo Hamburgo, Bresil | 30; NA; 73%; PWD; physical | Cross-sectional study with mixed methods | Bus | To identify ergonomics and accessibility issues faced by wheelchair users, and persons with mobility alterations when using public transport, from a user perspective. | On scale ranging from 0 (not satisfied) to 15 (satisfied)  Schedule: 5.36.  Employee training/ guidance: 6.24.  Waiting time for passengers: 5.41.  Service waiting time for wheelchair users: 6,06.  Distance to bus stop: 5.25.  Feeling of being a “burden to others: 5.84.  Ramp: 6.40.  Platform space: 6.06.  Space inside bus: 7.01.  Maneuverability space: 6.28.  Position in relation to door: 6.44.  Location of wheelchair user inside transportation vehicle: 6.57.  Belt: 7.18  Safety device at platform: 6.32.  Use of transportation in motion: 6.49.  Safety devices in transportation vehicle: 6.44.  Protection devices and handles: 6.58  Wheelchair dock inside bus: 6.27 Feeling of comfort in transportation vehicle: 5.55.  Position of legs in space provided: 5.94.  Positioning in space provided: 6.42 Stability at platform: 6.72.  Stability with the vehicle in motion: 5.27.  Design: 5.66. |
| Asplund et al., 2012 (42) | Umea, Sweden | 822; 71; 49%; PWD; physical, intellectual, visual, auditory, other | Cross-sectional quantitative study | Bus, train, flight, and ship | NA | Stroke survivors who had never used public transportation appear to overestimate the physical barriers and fear compared with those who had used it. |
| Bezyak et al., 2020 (11) | 50 states, United States of America | 1,748; 49; 38.5%; PWD; physical, visual, auditory,  speech/communication, cognitive, intellectual, psychiatric, and  chronic health conditions | Cross-sectional quantitative study | Bus, paratransit, taxi, walk or roll, commuter/light rail, Uber or Lyft, personal vehicle, and bicycle | To understand the barriers and  supports to accessing public transportation and the impact on community participation. | 65.5% of participants noted issues that prevented them from using public transportation as much as needed; 47.6% PWDs reported not having the same access to public transit as others in their community; Female participants experienced reduced access to public transport compared to males. |
| Bezyak et al., 2017 (43) | New York, USA | 4161; 49; 40.8%; PWD; physical, Blind/low vision, Deaf/hard of hearing, communication disability, mental health disability, and other | Cross-sectional quantitative study | Bus, taxi, light train, subway, paratransit | To provide  a full description of barriers experienced by individuals with disabilities when using the public transportation and the  complementary paratransit services. | Proportion of PWD who encountered barrier are presented below:  Drivers not calling out stops 30.2% Inappropriate driver attitude 26.7% Drivers lack of knowledge 23.0% Inability to navigate public system 20.1% Inaccessible stop/station 19.8% Gap or step to vehicle 18.9% Problems with lifts 17.2% Vehicle full 14.7% Drivers refuse to stop 14.1% Unable to secure wheelchair 7.6% Service animal problems 5.6% Wheelchair is too big/heavy 3.3% |
| Bigby et al., 2017 (25) | Victoria, Australia | 21; NA; NA; PWD; communication disabilities (autism, cerebral palsy, intellectual disability with cognitive and speech conditions, Intellectual disability with only cognitive conditions, acquired brain injury not related to stroke, deaf/hearing, and blind) | Cross-sectional qualitative study | Train | To investigate the experiences of people with communication disabilities on a train service to identify their perspectives about barriers and facilitators of accessibility. | Participants with physical, sensory, or speech impairments but without cognitive or visual impairments generally found information about the train system accessible, easy to use, and helpful. Visual announcements were inaccessible to people who were deaf or hard of hearing. At stations, high levels of noise created difficulties for staff to understand people who used speech generating devices to get their message across. Poor platform lighting made station names inaccessible when train windows were tinted, especially at night. Platform changes constituted a barrier for passengers who had physical disabilities. Participants wished to be regarded as similar to other passengers but to also have their need for assistance recognised and met without being singled out as different. |
| Broome et al., 2013 (50) | Hervey Bay and Brisbane, Australia | Study 1:  335; ≥60; 27.4% from Hervey Bay and 26.7% from Brisbane before intervention; 29.6% male from Hervey Bay and 28.9% male from Brisbane after intervention  Study 2: 100; 72.4; 22.9% for Hervey Bay group and 19.2% for Brisbane, Older adults | Longitudinal before-after quantitative study | Bus | To evaluate the impact of implementing age-friendly guidelines for public buses on bus use, useability, and social participation for older people. | Poor vehicle accessibility (primarily  difficulty getting on and off the bus). Distance to and from the bus stop.  Unsatisfactory timetables and scheduling (commonly characterised by infrequent buses). |
| Carlsson, 2004 (26) | Helsingborg and Kristianstad, Sweden | 20; 77; 15%; PWD; physical, visual, auditory | Cross-sectional qualitative study | Bus | To explore usability problems in public transport in a travel chain perspective focusing on the physical environment and inherent aspects of it. | Participants reported the following barriers:  At entrances: high steps, manual door-opening, round stairs, handrails only at one side, non handrails.  At the Public outdoor environment: long distance, too narrow pathway, branches hanging pathway, irregular walking surface, small holes, no kerb cuts, poor design of kerb cuts, difference in levels, steep side gradient on pathway, gradients, on light-contrast in surface, combined, combined pedestrian and cycle-way, grey posts on pathway, crossing with traffic light but no auditory signal, too short time for green, slippery white lines, traffic from two directions, limited light sight near a crossing seats, high seats, seats slope backwards, seats without back, back on the seat slopes backwards. On the Bus stops: cycle-way adjacent to the bus stop, narrow bus stops, no wither protection, grass on bus stop, no seats, low seats, high seats, without back, back of the seat slopes backwards, many buses stop at the same bus stop, timetable lacking, small text on timetables, glitter in the monitor, wrong information, difficult to interpret info, information table far away, no information about buses stopping at the bus stop. In the buses: bus doors swing out, bus not kneeling, bus stop far from the platform, stop at other places than the bus stops, no ramps, steps into the bus, steps inside the bus, too short time to enter and alight, no contrast making, all seat occupied, no handrails at seats, narrow space between seats, narrow space to the seat in front, no space in front of the seat for rollator, no space for the rollator, rear facing seat, tip-up seat, no use of handrails in the bus when using rollator/two sticks, bell-push-wrong place, information not possible to see at rearfacing seat, bus stop information lacking, only visual information, wrong information on bus stop information. |
| Casey, 2013 (27) | Dublin, Ireland | FGD:13; 41; 53.8%; PWD; visual  In-depth interviews:  2; 30 and 50; 0%; healthy persons | Cross-sectional qualitative study | Bus, train | To examine the experiences of people with sight loss in using public transport and  to explore the specific concerns that this group have. | Barriers concerned: lack of confidence in the staf, difficulties in stopping the bus (due to the extent of their sight loss), difficulties in gaining both accurate and accessible information prior to travel, difficulties in identifying bus numbers (due to the extent of their sight loss), lack of reliable audible announcements on both trains and buses.  Facilitators included awareness of the staff on visual disability, mobility training, colour contrasting grab rails, kneeling buses, braille on bell-pushes, reduction of steps on and off vehicles, and audible announcements. |
| Enginöz and Savli, 2016 (28) | Istanbul, Turkey | 12; NA; NA; PWD; physical and visual | Cross-sectional qualitative study | Metro | To examine the approaches on barrier-free design and level of accessibility at metro stations in Turkey. | Insufficient signboards; no braille signboard, and long distance at arrive to entrance of metro station; handrails without contrasting colour, and numbers and letters in embossed and braille alphabet on stairs, escalators and elevators; no braille and embossed warning and informative signboards at the height of 120-160 cm in the main hall and circulation areas; guiding paths for visually impaired people partially fixed at metro station; no signboard to guide to the accessible toilets. |
| Frost, Bertocci and Smalley, 2020 (48) | Louisville, USA | 384; ≥18; 44.3%; PWD; physical | Cross-sectional study with mixed methods | Bus | To solicit feedback on ramp related incidents and difficulties from WCUs who are reliant on transit bus ramps for safe and independent access to public transportation. | Nearly 4 out of 5 respondents (78%) experienced a ramp-related ingress or egress incident. 78% of wheelchair users (WCUs) had ≥ 1 ramp incident over the past 3 years, occurring during ingress (22%) and egress (18%). Of those, 22% were injured or had damage to their wheelchair.  Percentage identifying contributing factors:  - Steep ramp slope: 56%  - External threshold: 71%  - Wet ramp surface: 58%  - Ramp width: 64%  - Edge barriers: 38%  - Interior threshold: 88% |
| Hess, 2009 (44) | New York (NY) and California (CA), USA | 175 (NY) and 286 (CA); 76 (NY) and 77 (CA); 33% (NY) and 41% (CA); Older adults | Cross-sectional quantitative study | Bus, train | To explore associations  between older adults who do and do not ride fixed- route public transit and their neighborhood walking access to buses and trains. | Perceived barriers:  - Knowledge (mean, SD): 1.32 (0.83) in Buffalo, 1.64 (0,74) in San José  - Transfer (mean, SD): 1.67 (0.80) in Buffalo, 1.85 (0.73) in San José  - Physical barriers (mean, SD): 0.79 (0.29) in Buffalo, 0.67 (0.33) in San José  - Composite perception (mean, SD): 2.58 (0.46) in Buffalo, 1.95 (0.39) in San José  Greater walking distance to transit reduces the frequency of riding transit.  Each additional 5 minutes of walking distance to a transit stop or transit station reduces the frequency of riding transit by 9% for women and 8% for men ; In Erie County (Buffalo) snow is barrier for 60.3% of respondents; In San José, rain is barrier for 55.6% of respondents, and darkness is a barrier for 53.1% of respondents; In Buffalo, the perceived walking time: 8.3 minutes for frequent transit riders, 7.7 minutes for infrequent transit riders, and 8.0 minutes for non-transit riders. In San José, the perceived walking times to transit for infrequent transit riders and for non-transit riders are similar (about12.5 minutes) and are greater than the perceived time reported by frequent  transit riders(10.6minutes). |
| Hidalgo et al., 2020 (29) | Bogota and Medellın, Colombia  Santiago de Chile, Chile | 75; NA; NA; PWD (physical, visual, cognitive, auditory, and temporal), older adults, and healthy persons (caregiver) | Cross-sectional qualitative study | Bus, train | To identify barriers in public transport accessibility and travel experience. | Obstacles in sidewalks; discontinuities and lack of guiding infrastructure (dots and lines in the sidewalks); lack of space for circulation and ramps; unsafe intersections; lack of traffic lights with sound for the hearing impaired, or very short signal phases; Inadequate lighting; poor condition of the circulation surface; difficulties in accessing the stations; Prevalence of street vendors encroaching pedestrian spaces; lack of integration with bike paths; crowded platforms; lack of furniture to rest; lack of rain protection; inadequate access/egress buses/trains; lack of web and mobile applications for PWD; turnstiles lack sound modules to provide information on the ticket cards balance; Insufficient or dysfunctional voice and visual announcements on routes and next stops; Insufficient information about routes, their frequencies and schedules oriented to PWD; lack of information about accessible vehicles; In the case of buses, driving behavior causes sudden brakes and acceleration causing  discomfort to users; lack of protocols and adequate staff training to assist PWD; lack of special orientation in operating protocols to attend PWD; inadequate management of queues for farecard sales/recharge; insufficient provisions or information for recharging cards outside of stations; incorrect use of preferential spaces for PWD within buses and trains; lack of training of drivers and other systems users on the needs of PWD, resulting in lack of respect; Lack of preferential access for PWD to farecard sales/recharge/validation and areas within buses and trains, difficulty to accessand exit because of crowdedness. The average overall satisfaction with the public transport systems was 3.8 for Santiago, 3.2 for Bogota´ , and 4.0 for Medellın on a Likert scale ranging from 1 (very dissatisfied) to 5 (very satisfied). |
| Jahangir et al., 2022 (30) | Dhaka, Bangladesh | 30; ≥60; 83,3%; older adults | Cross-sectional qualitative study | Bus | To investigate key barriers in accessing buses in Dhaka and the consequences of these barriers to the everyday mobility of older adults. | Bus stops extremely busy during peak hours. Barriers occurring when getting into the bus due to overcrowding and many people hanging on the entry of the bus; Barriers while traveling on buses: few seats available for older adults. Intangible barrier: fear of being harassed inside the crowded buses; Other barriers: long waiting time. |
| Logan, Dyas and Gladman, 2004 (31) | Nottingham, UK | 24; 71; 71; PWD; physical | Cross-sectional qualitative study | Bus | To understand better the experiences, attitudes and beliefs of people with disabling illness to using transport. | Participants were afraid of injury or embarrassment from falling; 46% wanted to use transport but had lost their confidence; 20% did not know how to use buses. Participants felt happier going out in the summer than the winter, and avoided rain, ice, snow, wind and the dark. |
| Mah and Mitra, 2017 (49) | Oakville, Canada | Survey questionnaires:  131; 65; 22.3; older adults  In-depth interviews: 16; ±75; 25%; older adults | Cross-sectional with mixed methods | Bus | To explore older adults’ travel behaviour and experiences related to a recently introduced free transit program. | Survey shows that older adult with lower income (i.e., <CAD 40,000) was 5.34 times more likely to be impacted if the program stopped, compared to those with higher incomes (≥CAD 40,000). Partially-Adjusted OR (95% CI) =5.34 (1.61–17.67) with p-value≤0.05 In-deph interviews reveals: Bus drivers are helpful, polite and courteous and consequently motive older adults toward using free transit for senior (FTFS) program; FTFS program facilitates the spontaneous social interaction by meeting new people on the bus; FTFS program allows older adult to save money. |
| Mandhani, Nayak, and Parida, 2023 (45) | New Delhi, India | 254 (148 older adults, and 106 PWD); >59 (older adults) and 15-49 (PWD); 73% (older adults) and 63,2% (PWD); older adults and PWD; physical | Cross-sectional quantitative study | Metro station | To establish interrelations among service quality factors and identify service  improvements for older adults and physically disabled passengers using satisfaction data from these passengers of Delhi Metro. | Based on a 5-point Likert scale ranging from 1 (highly dissatisfied) 5 (highly satisafied), older adults and PWD are moderately satisfied with metro services (mean satisfaction values > 3). They are mostly satisfied with lighting in the  metro system, smart card facility, and cleanliness inside metro, and least satisfied with convenience at  metro stations, seat availability at metro stations, and ease of interchange within stations. Mean  satisfaction of older adults and PDW passengers with overall level of service and overall satisfaction with the metro trip are found to be 3.87 and 3.71, respectively. |
| Ogawa et al., 2020 (51) | Japan | 21; 67.4; 33.3%; PWD; Physical | Longitudinal before-after quantitative study | Bus, train | To identify the effects generated by patients with physical disabilities undergoing recovery rehabilitation to receive hands-on training in the use of public transportation, particularly regarding how possible changes in self-efficacy and perceptions of occupational enablement might be affected. | Self-efficacy after the intervention was higher than that before. |
| Olawole and Aloba, 2014 (47) | Osogbo,Osun State, Nigeria | 250; ≥60; 51.2%; older adults; | Cross-sectional quantitative study | Mini-bus (korope), taxi, motorcycle | To provide insight into the mobility characteristic and commuting patterns of the elderly and examine the socio-economic characteristics of the elderly; their perception of the quality of transport services and problems associated with the use of public transport services. | The mean satisfaction of the elderly, by age group (four groups), with transport  services ranges between M=2.34 and M=2.79. A moderate level of satisfaction (M=2.79, SD=1.84) with transport services is computed for the elderly in the age group 70–74 years and M=2.66, SD=1.59 for those above 75 years. |
| Park and Chowdhury, 2018 (32) | Auckland, Dunedin, Wellington, Christchurch, and Whanganui, New Zealand | 32; ≥15; 31.3; PWD; Physical and visual | Cross-sectional qualitative study | Bus, train | To investigate the barriers in a typical journey chain and provides the similarities and differences in the key barriers perceived by people with physical and visual impairments. | Number of times barrier is mentioned by participants:  -Long distance to/from stop/station (8).  -Footpaths (21).  -Urban environment (18).  -Construction (5).  -Crossings streets/road (3).  -Information (13).  -Lack of accessible parking at terminals (5).  -Wet Weather (3).  -Poor quality of service (10).  -Terminals and stops (13).  -Bus driver attitude and unawareness (26).  -In-Vehicle facilities (14).  -Other (3). |
| Putranto and Putri, 2018 (33) | Jakarta, Indonesia | 13; 38-53; 84.6%; PWD; visual | Cross-sectional qualitative study | Bus, train | To understand the satisfaction level of the blind on urban transportation system. | Staff was not knowledgeable regarding the needs of the blind (e.g., how to assist the blind to board and to alight the bus); they complained that the time required to get ticket was too long; the distance to reach Transjakarta bus stop was too long; not all the sidewalks installed with guiding and warning blocks. |
| Pyer and Tucker, 2017 (55) | Midlands and South, East England, UK | 69; 13-17; PWD; Physical | A multi-method approach: semi-structured interviews, video-tours and self-directed photography | Bus, train, taxi, car | To explore the layers of disadvantage experienced by teenage wheelchair users in their attempts to use public and private transport to access leisure spaces. | Lots of buses have steps and the use of public transport often signified worry and fear. At certain railway stations there is only one lift (If it is not working then it is impossible to get off at certain platforms). |
| Rambaldini-Gooding et al., 2022 (34) | Wollongong, Australia | 13, >60, 61,5%, older adults | Cross-sectional qualitative study | Bus, bus stop | To explore older people's use of free bus service. | All the buses had identified priority seating and grab rails, creating potentially safe spaces for older people.  One participant, who had a walking stick, described how the drivers were ‘good’ and lowered the bus to enable his safe boarding.  During the trip, priority seating, wheelchair spaces and a low floor at the seats were left vacant for older people. Other passengers were courteous towards older people.  Older people identified the health and well-being benefits that they got from using the bus service.  The bus service promoted physical activity for participants. A male participant stated that ‘the bus system overall is great. It stops us being stagnant.  The bus service was observed to be popular with older people during daytime travel and identified as important for participants accessing the hospital, pharmacies and the library.  Participants also reported using the service to maintain connections with family and friends. |
| Ravensbergen, Newbold and Ganann, 2022 (35) | Hamilton, Canada | 24; ≥65; 38%; older adults | Cross-sectional qualitative study | Bus | To explore older adults’ experiences of becoming public transit users through a self-efficacy lens. | The lack of confidence was associated with the lack of experience. Development of knowledge and skills in riding public transit improved confidence. |
| Ravensbergen et., 2021 (36) | Hamilton, Canada | 24; ≥65; 38%; older adults | Cross-sectional qualitative study | Bus | To explore older adults' experiences using public transit with a focus on the mobility work. | Barriers experimented were bad weather conditions (winter conditions); long walking distance to and from the bus stop; negative attitude of bus drivers; standing on a bus while it is in motion; stress generated by social expectation to be quick as healthy persons; step onto or off the bus |
| Remillard et al., 2022 (37) | Atlanta, USA | 60; 69; 42%; PWD; physical | Cross-sectional qualitative study | Bus, train or subway, taxi, Uber, or Lyft | To provide detailed insights on transportation challenges experienced by Persons aging with mobility disability  along with recent Federal programmatic initiatives designed to enhance access and mobility for transportation for older adults and people with disabilities. | Participants reported barriers such as lack of availability of transportation, steps and stairs, safety, financial limitations, wheelchair access and storage, lack of handicap accessible spaces and social attitudes when riding train/subway or taking a bus. |
| Risser et al., 2015 (17) | NA, Sweden | 34 articles with various sample sizes; NA; NA; PWD; cognitive disability | Qualitative systematic review | bus, tramway, train | To identify and summarise scientific literature focusing on people with cognitive impairments and their needs in  public transport, applying a travel chain perspective. | Barriers identified in included studies are:  -Lack of both assistive devices and trained personnel helping and assisting with  orientation.  - Access to and use of public transport is dependent on the ability to balance internal (i.e., knowledge, ability and willingness to use public transport) and external factors (i.e., accessible and usable infrastructure, and personal support from staff of infrastructure service providers).  - People with cognitive impairments often have problems related to orientation and navigation  - The pedestrian environment must also be well-maintained, be even in level and constructed in a material that give a flat paving in order to facilitate outdoor mobility  - Information must be easily provided and understood, for instance that displays presenting timetables  - The tendency of automation in public transport systems can have negative effects in critical situations when personal support is needed |
| Stjernborg, 2019 (38) | Stockholm, Sweden | 389 (complaints); NA; NA; PWD; physical | Cross-sectional qualitative study | Bus | To provide an overview of the character of complaints concerning accessibility  left to a public transport provider. | Barriers bundled in courtesy, closeness to the curb when stopping the vehicle, and accessibility ramps are: lack of knowledge on the use of ramp access, Ramp out of order, Refusal to deploy the ramp, lack of courtesy from drivers, bus not lowering Barriers related to elevator/escalator are: elevators or out of service or broken, and stairs lacking ramps in stations; Other barriers related to timetables, escort service, seating, and other Issues are: No automatic subscription to printed schedules, No escort service, misunderstandings  between the escort and the traveller, insufficient lighting both on boar vehicles and in stations, and too low seats. Help from other passengers to support PWD getting on or off the vehicles due to accessibility equipment not being used |
| Tennakoon et al., 2020 (39) | Colombo district, Sri Lanka | Four FGD (step 1):  NA; ≥12; NA; PWD; physical, sensory, learning impairment  Four FGD (step 2):  NA; ≥60; NA; Older adult | Cross-sectional qualitative study | Bus, three-wheeled taxi, railway | To understand the barriers and facilitators for safe and  accessible transportation from the standpoint of older people and people living with disabilities who are resident in an urban area of Sri Lanka. | Participants felt the physical dimensions of the road infrastructure, particularly pedestrian environments, not safe.  Roads had a busy atmosphere with too many people and too many vehicles.  The lack of sidewalks and pavements, or their narrowness, were key risks for safe transportation.  The inaccessible design of public buses, trains and stations was a significant barrier to safe transportation which created physical and emotional discomfort for all participants; The physical location of bus-stops was an issue for some participants.  One older female participant felt the bus-stop inconveniently located in front of a bar and next to the petrol station, which meant the area was crowded at all times.  Participants described observing breaches of road rules including speeding, weaving in and out of lanes, and running through road crossings.  PWD emphasised that they did not need any sympathy from the public but an understanding of their situation.  Participants felt embarrassed about the harsh behaviours of service providers and public and were mentally exhausted due to fear of injury, feelings of anxiety, insecurity and powerlessness.  Physical and financial dependency were barriers to safe and accessible transportation.  The obstacles on the pavement and unevenness were felt particularly dangerous by people with sensory and physical disabilities. Participants with visual impairments were concerned about audible signalling systems and pavement quality.  Participants with hearing impairments emphasised educating the public about basic sign language |
| Unsworth et al., 2019 (18) | NA, Australia | 26 articles with various sample size; NA; NA; PWD; physical | Systematic review of the peer-reviewed literature | Bus, tram, light train | To investigate literature on public transport access for people using mobility devices, excluding transit restraint and securement literature. | Four studies focused on uneven pavement surfacing, lack of dropped curbs, steps instead of ramps, narrow doorways, controls for pedestrian lights or lift access that are too high, badly designed street displays. Three studies highlight information placed out of reading reach, inappropriate spaces for wheeled mobility devices,  long waiting times, little shelter. Three studies point out drivers' attitude.  Five studies underline ramp design, concerns with ramp angle and deployment.  Four studies point out insufficient space for wheelchair |
| Velho, 2019 (40) | London, UK | 34; ≥18; 47%; PWD; physical and healthy persons | Cross-sectional qualitative study | Bus, train | To describe the barriers  faced by wheelchair users in the transit network (from physical to social) and the largely negative emotions that these barriers bring out on these passengers. | The use of buses is a source of negative emotion such as stress, anxiety, frustration, annoyance, depression, misery, worthless, lack of confidence or courage or bravery for WCUs living in London. |
| Velho et al., 2016 (54) | London, UK | Pilot study:  4; NA; 100%; PWD; physical  Engineering component:  7; NA; 100%; PWD; physical | Longitudinal mixed method | Bus | To research the barriers faced by wheelchair users in public transport to establish the breadth of issues faced by wheelchair users. | Technological barriers: Broken ramp when deploying ramps; Public humiliation that siren noise seems to generate when deploying ramp.  Biomechanical barriers:  - Climbing the 6.5% incline people used significantly greater force than level propulsion (106.90N vs. 50.36N) and climbing the 12% incline people used a significantly greater force than climbing the 6.5% incline (139.63N vs. 106.90N)  - During the 6.5% incline, peak shoulder forces were over two times body weight and during the 12% incline task, peak shoulder forces were over three times body weight.  Spatial barriers: Priority space often occupied or insufficient within the bus for wheelchairs users; the size of the wheelchairs.  Social barriers: debate between wheelchairs vs buggies resulting from space problems within buses; drivers do not stop the bus at all for a wheelchair user to board it; negative reactions towards wheelchair users; verbal and physical abuse |
| Verbich and El-Geneidy, 2016 (52) | Verbich and El-Geneidy 2016 | 16.830 (regulars riders), 4.136 (encumbered riders), and 555 PWD; ≥16; NA | Explanatory descriptive study | Bus | To uncover how diverse  aspects of bus services can differentially impact satisfaction of different riders. | Satisfaction of disabled riders with overall service: 90%  - Overall satisfaction of disabled riders is only influenced by information at the stop/shelter  - Having a seat does not influence the satisfaction of riders with disabilities, and since most riders with disabilities had a seat, our models demonstrate the effectiveness of priority seating as a policy  - Satisfaction of riders with disabilities depends on information availability at the bus stop (increasing by 1.02 times [Odd ratio] for PWD (p<0.05)), as well as trip speed and reliability (increasing by 1.03 times [Odd ratio] for the same population (p<0.01)). |
| Wayland et al., 2022 (41) | New South Wales and Victoria, Australia | 26; 18-28; 46.1%; PWD; Physical, visual, intellectual, hearing, other conditions (lupus, schizophrenia down syndrome, etc.) | Cross-sectional qualitative study | Bus and train | To explore the experiences of young adults with disabilities  regarding their perceptions of interpersonal discrimination  on public transport in two Australian states. | One participant identified the discourteous behaviour by bus drivers towards PWD as a discriminatory act.  Another participant describes frequent and significant discriminatory experiences including unwanted physical assistance and verbal and sexual assault. The same participant pointed out the lack of education of other passengers about health concerns that PWD can have, leading them to regard the PWD as strange people.  Another participant reported the refusal of some bus drivers to let PWD disembark at desired or called stops. Moreover, he argued that the judgmental and reductive comments made by young passengers towards PWD on the train are discriminatory behaviour. |
| Wong et al., 2017 (53) | Hong Kong, China | 613; ≥60; 48.4%; Older adults | Explanatory descriptive study | Bus, railway, public light bus, taxi | To evaluate Elderly uses' level of satisfaction with various public transport modes. | Seat availability, travel stability, driver's attitude, ease of boarding and alighting, travel time and reliability and the condition of stations or stops influence satisfaction with public transport use at 1% levels; wait time for service influences satisfaction with public transport use at the 5% levels; Internal temperature and walking distance to stations or stops do not so. |
| NA: Not available; PWD: people with disabilities; FGD: fucus group discussion | | | | | | |
